# Single Cell Transcriptomics of Paired CSF and Blood Immune Populations Reveals Unique Macrophage Population with Relevance to Alzheimer's Disease

**DOI:** 10.1002/alz70855_097563

**Published:** 2025-12-23

**Authors:** Beth Stevens, Samuel E Marsh, Rebecca Fulthorpe, Sarah Murphy, Tushar Kamath, Vahid Gazestani, David Lieb, Thomas Eisenhaure, Vishal Sarsani, Miguel Reyes, Johan Gobom, Clary Clish, Henrik Zetterberg, Ville Leinonen, Evan Macosko, Nir Hacohen

**Affiliations:** ^1^ Boston Children's Hospital, Boston, MA, USA; ^2^ Broad Institute of MIT and Harvard, Cambridge, MA, USA; ^3^ The Broad Institute of MIT and Harvard, Cambridge, MA, USA; ^4^ Broad Institute, Cambridge, MA, USA; ^5^ University of Gothenburg, Mölndal, Sweden; ^6^ Kuopio University Hospital, Kuopio, Finland; ^7^ University of Eastern Finland, Kuopio, Finland

## Abstract

**Background:**

Alzheimer's disease (AD) is a neurodegenerative condition with etiology firmly rooted in immune dysregulation. While genetic risk is increased in microglia, the brain's resident myeloid cell, myeloid cells throughout the body show high enrichment for disease risk genes as well. The role of peripheral immune cells in AD pathogenesis is relatively unknown, however, and may play important roles in waste clearance. Leveraging a cohort of idiopathic normal pressure hydrocephalus (iNPH) patients from which we have paired brain, cerebrospinal fluid (CSF), and peripheral blood monocytes (PBMCs), we have investigated cross‐compartment transcriptional changes to better understand how peripheral immune function is altered in AD.

**Method:**

We have performed scRNA‐seq on paired samples (blood PBMCs, brain biopsy, and CSF, *n* = 100), from iNPH patients, ∼40% of which have early stage AD, to understand neuroimmune changes. We have further performed a large‐scale integrative analysis across previously published CSF scRNAseq datasets (>200 patients and 400,000 cells).

**Result:**

Our analysis has found a unique population of macrophages present in the CSF that are distinct from CNS microglia and border macrophages, and peripheral monocytes. These cells are abundant in the CSF of all samples. Strikingly, we find that these cells exhibit enrichment of polygenic Alzheimer's disease heritability, including very high expression of the two most significant AD genetic variants, APOE and TREM2. Furthermore, we find that these macrophages display altered gene expression profiles in patients with AD pathology or clinical AD diagnosis, underscoring the importance of understanding these enigmatic cells. To deepen our understanding of CSF macrophages, we have also performed large‐scale integrative analysis with previously published CSF scRNA‐seq datasets. We identify this macrophage population across all datasets, and are working now to understand disease‐specific variations in CSF macrophage state.

**Conclusion:**

By profiling the immune cells present in the CSF and comparing them to immune cells in the blood, we have identified a population of CSF macrophages that are unique to the CSF that show heritability enrichment for AD risk genes. These immune cells may play critical scavenging and clearance roles in disease and future work will clarify their role in AD.

